# SEOM clinical guidelines for diagnosis and treatment of glioblastoma (2017)

**DOI:** 10.1007/s12094-017-1763-6

**Published:** 2017-10-30

**Authors:** M. Martínez-Garcia, J. Álvarez-Linera, C. Carrato, L. Ley, R. Luque, X. Maldonado, M. Martínez-Aguillo, L. M. Navarro, M. A. Vaz-Salgado, M. Gil-Gil

**Affiliations:** 1 Oncología Médica, Hospital del Mar-Parc de Salut Mar, Barcelona, Spain; 2 Neuro-radiología, Hospital Ruber Internacional, Madrid, Spain; 3 Anatomía Patológica, Hospital Universitari Germans Trias i Pujol de Badalona, Barcelona, Spain; 4 Neurocirugía, Hospital Ramón y Cajal, Madrid, Spain; 5 Oncología Médica, Complejo Hospitalario Universitario de Granada Virgen de las Nieves, Granada, Spain; 6 Oncología Radioterápica, Hospital Universitari Vall d’Hebron, Barcelona, Spain; 7 Oncología Médica, Complejo Hospitalario de Navarra, Pamplona, Spain; 8 Oncología Médica, Hospital Universitario de Salamanca-IBSAL, Salamanca, Spain; 9 Oncología Médica, Hospital Ramón y Cajal, Madrid, Spain; 10 Unidad de Neuro-oncologia. Oncología Médica Institut Català d’Oncologia (ICO)-Hospital Universitari de Bellvitge IDIBELL L’Hospitalet, C/de la Feixa Llarga, s/n, Hospitalet de Llobregat, 08907 Barcelona, Spain

**Keywords:** Glioblastoma, Treatment, Diagnosis, Guidelines

## Abstract

Glioblastoma (GB) is the most common brain malignancy and accounts for over 50% of all high-grade gliomas. Radiotherapy (RT) with concomitant and adjuvant temozolomide (TMZ) chemotherapy is the current standard of care for patients with newly diagnosed GB up to age 70. Recently, a new standard of care has been adopted for elderly patients (≥ 65 years) based on short course of RT and TMZ. Several clinically relevant molecular markers that assist in diagnosis and prognosis have recently been identified. The treatment for recurrent GB is not well defined, and decision-making is usually based on prior strategies as well as several clinical and radiological factors. The presence of neurologic deficits and seizures can significantly impact quality of life.

## Introduction

Glioblastoma (GB) is the most common and the most aggressive primary brain tumor with an incidence of 3–5 cases per 100,000 inhabitants per year and a slight predominance in males. 4000 new cases of malignant gliomas are diagnosed each year in Spain, from which more than one-third are GB [1]. GB may develop at all ages, with the peak incidence in the sixth decade of life; and the mean age at diagnosis of 62 years. Most GB arise ‘de novo’, whereas, secondary GB develop from lower grade glioma.

Exposure to ionizing irradiation has been associated with increased risk of development of glioma, while the association with the use of cell phones has not been confirmed so far. Rare hereditary syndromes confer an increased risk for glioma such as neurofibromatosis type 1, Cowden, Turcot, Lynch and Li-Fraumeni syndromes.

The aim of these guidelines is to summarize current evidence and to give evidence-based recommendations for clinical practice to medical professionals of all disciplines involved in the diagnosis and care of patients with GB.

## Methodology

This SEOM Guideline has been developed with the consensus of ten physicians from different specialties with dedication to neuro-oncology. Five of them were chosen by the Neuro-Oncology Research Spanish Group (GEINO), other five by the Medical Oncology Spanish Society (SEOM). We decided to use the US Agency for Healthcare Research and Quality Service Grading System (USPSTF) to assign a level of evidence and a grade of recommendation to the different statements of this guideline (Table 1) [2].

**Table 1:** Levels of evidence and grades of recommendation according to US Agency for Healthcare Research and Quality Service Grading System (USPSTF)

Levels of evidence	
I	Evidence from at least one large randomized, controlled trial of good methodological quality (low potential for bias) or meta-analyses of well-conducted randomized trials without heterogeneity
II	Small randomized trials or large randomized trials with a suspicion of bias (lower methodological quality) or meta-analyses of such trials or of trials with demonstrated heterogeneity
III	Prospective cohort studies
IV	Retrospective cohort studies or case–control studies
V	Studies without control group, case reports, expert opinions
Grades of recommendation	
A	Strong evidence for efficacy with a substantial clinical benefit, strongly recommended
B	Strong or moderate evidence for efficacy but with a limited clinical benefit, generally recommended
C	Insufficient evidence for efficacy or benefit does not outweigh the risk or the disadvantages (adverse events, costs, etc.), optional
D	Moderate evidence against efficacy or for adverse outcome, generally not recommended
E	Strong evidence against efficacy or for adverse outcome, never recommended

## Guide recommendations

### Clinical diagnosis and initial assessment

GBs are infiltrating tumors that appear as space-occupying lesions, which dissemination usually remains limited to the nervous system. The symptomatology of GB depends on its location. The most frequently presenting symptoms are headache, seizures, and motor and/or sensory disturbances).

Initial assessment ideally should include magnetic resonance imaging (MRI) (II, B) [3, 4]. Contrast agent dose and composition are also critical to achieve precise and reproducible serial measurements. Optimal contrast is 0.1 mmol/kg or up to 20 cc dose injection with a gadolinium-chelated contrast agent. The most effective window for acquiring post contrast T1WI is between 4 and 8 min after administration. It is recommended (if perfusion techniques are not obtained) to acquire T2WI after injection and just prior to post contrast T1WI as T2WI [4]. Advanced MRI sequences include perfusion [cerebral blood volume (CBV)/permeability], diffusion (diffusion-weighted imaging/ADC) and proton magnetic resonance spectroscopy (MRS). They provide relevant data related to hemodynamic, cellular, and metabolism and help to identify glioma subtype and aggressiveness (III, C).

Evaluation and clinical decision-making in GB patients should be based on recommendations from multidisciplinary tumor boards.

### Surgery

Surgery represents the first therapeutic approach, reduces mass effect and obtains tissue for diagnosis. Maximum safe resection (without compromising neurological function) produces survival benefits (II, C), [5, 6], however a threshold for the minimum extent of resection and maximum postoperative residual volume have yet to be established. The development of a new neurological deficit after surgery is associated with decreased overall survival (OS) [7]. Neuronavigation systems, intraoperative image studies with MRI or ultrasounds, fluorescence dye 5-aminolevulinic acid (5-ALA) improve the extent of resection and this last one resulted in an improved progression-free survival (PFS) (I, A) [8]. Intraoperative MRI and intraoperative cortical and subcortical mapping techniques have shown a safer total resection as well, but without an improvement in OS [9].

When resection is not feasible (due to location or extension of the tumor), a biopsy should be performed obtaining enough amount of tissue for molecular assessment. Elderly patients without major comorbidities tolerate aggressive surgery and have prolonged survival as compared with similar patients undergoing biopsy only.

Postoperative MRI must be performed during the 24–48 h after tumor excision (II, B) [4] to avoid radiological changes related to subacute hemorrhage, ischemia and inflammation that appear beyond 72 h. This MRI allows assessment of the extent of resection (part of the RPA prognostic classification) and is the baseline image for follow-up (I, B).

### Pathological assessment and molecular biomarkers

Histological evaluation is mandatory. GB diagnosis should be based on the criteria established by the World Health Organization (WHO) classification. GB is defined as an astrocytic infiltrating tumor with one or both, necrosis and microvascular proliferation. By definition, GB corresponds to a grade IV, having the worst prognosis among infiltrating gliomas [10].

Molecular biomarkers represent additional tools for diagnosis and treatment decisions, and are becoming part of the routine practice. Depending on the *isocitrate dehydrogenase* (*IDH*) gene mutation status, GB are divided into *IDH* wild type and *IDH*-mutated, tumors, with different prognosis (II, A). For *IDH* status analysis, WHO recommends immunohistochemical (IHC) determination of IDH1-R132H, the most frequent mutated form. For *IDH1* mutation-negative cases, if the patient is younger than 55 years, it is recommended to complete the study by sequencing both *IDH1* and *IDH2* genes. For patient ≥ 55 years, only those with a history of a preexisting lower grade glioma, those with midline location (in which “diffuse midline glioma, H3 K27M” has not been discarded) and those with known *ATRX* mutation should be sequenced. Methylation status of the promoter of *methylguanine methyl transferase* (*MGMT*) gene has been largely recognized as a predictive factor for alkylating chemotherapy in GB [11]. *MGMT* promoter methylation status can be assessed by different methodologies, pyrosequencing and methylatio-specific PCR being the most frequently used in clinical practice are (II, A).

### First-line treatment

Radiotherapy (RT) plus concomitant and adjuvant temozolomide (TMZ) showed, in a large randomized phase III trial, a significant improvement in median, 2 and 5 years survival and represents the standard treatment in patients between 18 and 70 years old (I, A) [12]. RT is administered to a total dose of 60 Gy in a fractionated localized planning, using a fraction of 1.8–2 Gy/day; 5 days/week, in a field that includes a 1–2 cm margin around the image pickup-defined contrast T1 or all of the abnormal volume defined on T2 or FLAIR image. TMZ is administered daily (75 mg/m^2^ day) for 7 day/week, during RT (6 weeks) and approximately 1 month after the completion of RT/TMZ, TMZ is given for five consecutive days every 28 days (150–200 mg/m^2^/day) for six cycles (I, A). There is no evidence from randomized studies to determine the benefit of prolonging chemotherapy beyond six adjuvant cycles for patients without disease progression. To enhance TMZ absorption fasting is recommended (1 h prior and minimum of 1 h after). The most common acute toxicity are: nausea and vomiting (antiemetic treatment is advised); neutropenia and thrombocytopenia (hematologic control is required) and lymphopenia (prophylaxis against pneumocystis is recommended, specially if chronic use of corticosteroids) (II, B) [5, 6, 12].

In elderly patients, (> 65 year-old) a phase III study has shown that hypofractionated RT (40 Gy/15 sessions) plus TMZ 75 mg/m^2^/daily followed by adjuvant TMZ, 12 cycles (5 days every 28 days at doses of 150–200 mg/m^2^/day) significantly improves both OS and PFS (I, A) [13]. In patients with *MGMT* methylation, OS was almost doubled with RT/TMZ (13.5 m) than with RT alone (7.7 m). In patients with unmethylated *MGMT*, no statistical significance (*p* = 0.055) was achieved; but patients treated with RT/TMZ had a clear tendency towards a better OS. The regimen was well tolerated and there were no differences in quality of life, thus this strategy can be considered the new standard of care for patients > 70 years (I, A). For fragile elderly patients with *MGMT* methylation, in which radiation therapy could have a negative impact in terms of toxicity, TMZ alone is an accepted approach (II, A) [14].

Two randomized trials have explored the role of the addition of bevacizumab (BEV) to standard RT/TMZ followed by TMZ. These studies have shown an improvement of 3–4 months in PFS, without impact in OS [15, 16]. Therefore, this drug has not been approved for this indication.

Tumor-treating fields (TTF) represents a new therapeutical strategy for GB. It delivers low-intensity, intermediate-frequency alternating electrical fields that exert selective toxicity in proliferating cells through antimitotic mechanisms. A phase III randomised study demonstrated a 2.9-month improvement in PFS and a 2.8-month improvement in OS with the addition of TTF to adjuvant TMZ after RT plus concomitant TMZ [17]. TTF has been approved by the FDA and EMA for newly diagnosed supratentorial GB (I, B), but due to low cost/benefit ratio, it has not been approved by most European countries.

Eventually, for patients with poor performance status (PS), the best treatment is supportive care.

### Follow-up

Outside clinical trials, the first follow-up MRI should be performed approximately 1 month after the completion of RT and then every 3 months unless otherwise clinically indicated. Patients should be scanned on the same MRI equipment during follow-up examinations or at least on the same field strength, to ensure minimal variability. The Response Assessment in Neuro-oncology Working Group (RANO) criteria is the recommended criteria for radiological assessment of high-grade gliomas. RANO takes into account signal change on T2/FLAIR sequences and the contrast-enhancing component of the tumor as well as clinical data and corticosteroid therapy status (see Table 2). In 2010, RANO specifically addressed the issue of the so called pseudoprogression (increased contrast enhancement on imaging 4–12 weeks after the end of RT and concomitant TMZ that maybe is due to reactive process and no real tumor progression). RANO criteria specify that, within the first 12 weeks after completion of RT, tumor progression can only be established if most of the new enhancement occurs outside the radiation field or if histologic confirmation of progression is obtained [18]. There is some evidence that pseudoprogression is more likely to occur in *MGMT*-methylated tumors [19]. Recently, the Neurologic Assessment in Neuro-Oncology (NANO) scale: has been published [20]. This is a tool to assess neurologic function for integration into the RANO criteria providing an objective clinician-reported outcome of neurologic function with high inter-observer agreement with potential use in clinical trials and in daily practice.

**Table 2:** RANO criteria

Criterion	CR	PR	SD	PD
T1 gadolinium	None	≥ 50% ↑	< 50% ↑ but < 25 ↑	≥ 25% ↑
T2/FLAIR	Stable or ↓	Stable or ↑	Stable or ↑	↑
New lesion	None	None	None	Present
Corticosteroids	None	Stable or ↑	Stable or ↑	NA^a^
Clinical status	Stable or ↑	Stable or ↑	Stable or ↑	↑
Requirement for response	All	All	All	Any

*CR* complete response, *PR* partial response, *SD* stable disease, *PD* progression disease, ↑ increase, ↓ decrease, *FLAIR* fluid-attenuated inversion recovery, *NA* not applicable

^a^Increase in corticosteroids alone will not be taken into account in determining progression in the absence of persistent clinical deterioration

### Recurrent glioblastoma

**Fig. 1:**
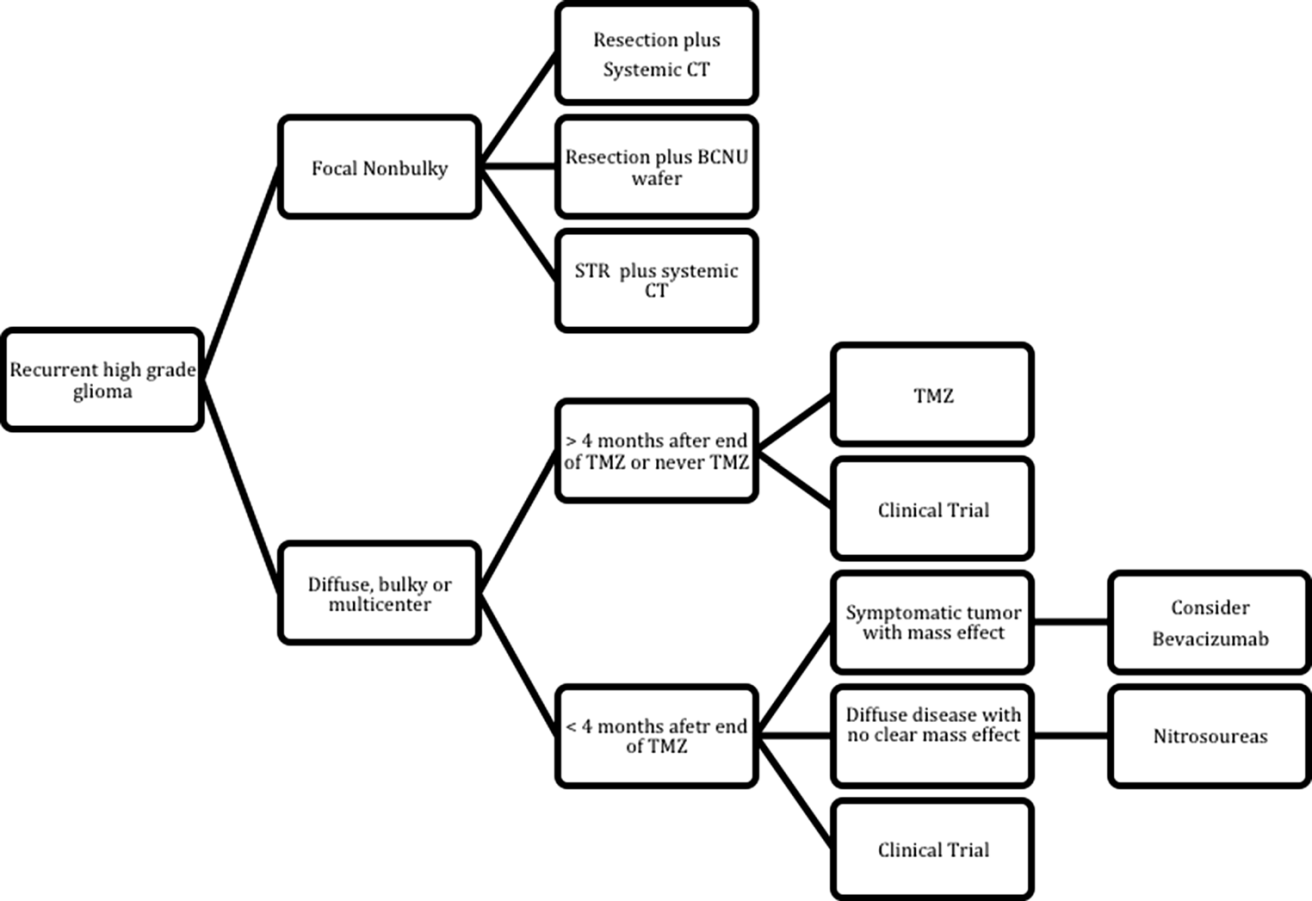
Treatment algorithm for glioblastoma in progression after RT. *CT* chemotherapy, *STR* stereotactic radiosurgery, *TMZ* temozolomide

A standard approach for recurrent GB has not been established. Several prognostic factors need to be taken into consideration to select the therapy, such as, tumor size and location, performance status and steroid requirements [21] (Fig. 1). The best option is the enrollment into clinical trials. If this is not an option, a second-line treatment should be considered.

#### Chemotherapy

The most widely used systemic agents include nitrosoureas, BEV and TMZ (see Table 3), but none of them is approved by EMA.

**Table 3:** Chemotherapy regimens commonly used in recurrent glioblastoma

Temozolomide	Conventional	150 mg/m^2^ (200 mg/m^2^ if no previous CT) × 5 days every 28 days
	Extended schedules	50 mg/m^2^/day continuous
		75–100 mg/m^2^ d1–d21 every 28 days
		150 mg/m^2^ for 7 days every 14 days
BCNU	200 mg/m^2^ iv every 6–8 weeks	
CCNU	100–130 mg/m^2^ po every 6 weeks	
Fotemustine	Addeo schedule	80 mg/m^2^ day 1, 15, 30, 45, 60 followed by a rest lost 4 weeks and a maintenance phase of 80 mg/m^2^ every 4 weeks
	Brandes schedule	75 mg/m^2^ days 1, 8 and 15 followed by a rest lost 5 weeks and a maintenance phase of 100 mg/m^2^ every 3 weeks
	Fabrini schedule	100 mg/m^2^ days 1, 8 and 15 followed by a rest lost 4–6 weeks and a maintenance phase of 100 mg/m^2^ every 3 weeks
PCV	Every 6 weeks	Procarbazine 60 mg/m^2^ days 8–21 CCNU 110 mg/m^2^ d1 Vincristine 1.4 mg/m^2^ days 8 and 29
Bevacizumab (BEV)	Monotherapy	10 mg/kg every 14 days
	Plus irinotecan (IT)	BEV 10 mg/kg + IT 125 mg/m^2^ every 2 weeks
	Plus CCNU	10 mg/kg every + CCNU 90 mg/m^2^ 14 days
	Plus fotemustine	75 mg/m^2^ days 1, 8 followed after 3 weeks arrest 75 m/m^2^ every 6 weeks
Carboplatinum	AUC 5 every 4 weeks	

Lomustine (CCNU) has shown a very modest improvement in OS (median 7.1–9.8 months), and it has been used as the control treatment arm in many studies. Fotemustine is an other nitrosourea that has proved activity in phase II studies in GB, with and adequate safety and tolerability profile.

**Table 4:** Summary of recommendations

General recommendations		Levels of evidence and grades
Karnofsky PS, neurological function, age, and degree of surgery are prognostic factors and need to be considered in clinical decision		I, A
The diagnostic imaging approach of first choice is MRI without and with contrast enhancement		II, B
The largest surgical removal is recommended; while preserving neurological function		II, C
BCNU wafer		II, C
If complete or partial resection, an MRI should be performed within 72 h after surgery		IV, B
Histological diagnosis is mandatory and should include sufficient tissue for molecular tumor characterization		IV, B
*MGMT* promoter methylation, gene (*IDH*) mutations are commonly determined		II, A
An apparent increase of tumor volume on MRI in the 1^st^ months after local therapeutic interventions (including RT and experimental local treatments) may reflect pseudoprogression		II, B
Newly diagnosed GB		
Age < 70 years or	RT (60 Gy in 30) plus concurrent TMZ, followed by adjuvant TMZ × 6 cycles	I, A
Age > 65–70 years	RT (40 Gy in 15) plus concurrent TMZ, followed by adjuvant TMZ × 12	I, A
Unfit > 65 years no methylated *MGMT*	Radiotherapy (50 Gy in 28 fractions)	II, B
Unfit > 65 years and methylated *MGMT*	TMZ alone	II, A
Recurrent GB		
PCV or single-agent nitrosourea therapy may achieve similar tumor control rates compared with TMZ		II, B
Bevacizumab: High response rates and better PFS but without differences in OS		I, B
TTFs failed to prolong survival compared with second-line chemotherapy		II, D
Re-irradiation (for small tumors)		IV, C
Reoperation (in particular patients with an acute mass effect) ± BCNU wafer		IV, C (surgery) II, C (BCNU wafer)

*PS* performance status, *MRI* magnetic resonance image, *RT* radiotherapy, *TMZ* temozolomide, *PCV* procarbazine CCNU and vincristine, *TTF* tumor-treating fields

Retreatment with TMZ could be an option for patients with failure beyond 4–6 months from the initial therapy [22] (II, B). Extended schedules were developed in over come TMZ resistance, unfortunately randomized studies have not shown superiority to standard dosing and produced greater lymphopenia [22, 23]. Combination of procarbazine, CCNU and vincristine (PCV schedule) may represent another alternative with similar activity to TMZ (II, B) [23].

Regarding antiangiogenic therapies, BEV has demonstrated encouraging efficacy in several phase II clinical trials in recurrent GB, leading to the approval of this drug by the FDA (I, B). However, the OS benefit of BEV in recurrent GB remains unclear [24]. A recent EORTC clinical trial, randomized 437 patients to BEV + CCNU versus CCNU, obtaining a significant difference in PFS (Median PFS: 4.2 m with de combination vs 1.5 m with CCNU; HR 0.49, CI 0.39–0.61), but no difference in OS (Median OS: 9.1 vs 8.6 m; HR 0.95, 0.74–1.21) [25].

Finally, in a phase III trial for recurrent GB, TTF failed to prolong survival compared with second-line chemotherapy (physician’s choice) (I, A) [26].

#### Salvage surgery

For recurrent GB, the decision of reoperation must be individualized and based on PS, age, and surgical feasibility (IV, C). There are no prospective data available on the impact of reoperation in OS. The most significant predictors of survival after reoperation are age, interval between surgery, PS, and ependymal involvement. Salvage surgery and implantation of carmustine-impregnated wafers may lead to marginal prolongation of survival compared with placebo (II, C) [27].

#### Salvage RT

There is a lack of prospective consistent data for re-irradiating recurrent gliomas. It could be used especially if long interval since prior RT and/or if there was a good response to prior RT. Based on retrospective patient series, repeat RT using modern high-precision techniques such as fractionated stereotactic RT may be an option for selected patients with good PS and small recurrent tumors (II, B) [21, 28] (Table 4).

### Supportive care and patient management

Patient management includes pharmacological interventions with corticosteroids, antiepileptics, analgesics, antiemetics and other measures such as psychological and social support. Corticosteroids are not necessary in patients without edema-associated neurological deficits or increased intracranial pressure. Dexamethasone is the preferred steroid for the treatment of vasogenic edema in symptomatic patients because of its low mineralocorticoid effects and long half-life, the lowest effective dose is recommended. Prophylactic use of antiepileptic drugs outside the perioperative period is not indicated (III, C) [29]. Levetiracetam is the better monotherapy option due to lack of interactions, easy dosing, oral and intravenous availability and fewer adverse effects. GB confers a special risk for thromboembolic events mainly in patients with reduced mobility or limb paresis, poor PS and steroid use. Anticoagulation remains underutilized in patients with GB, due to concerns of potentially intracranial bleeding. Retrospective studies indicate that anticoagulation can be safely used in GB patients and low molecular weight heparins are the treatment of choice [30]. Consider the use of a palliative care team for symptom management at end of life [31].
